# Evaluation of Host Immune Response in Diabetic Foot Infection Tissues Using an RNA Sequencing-Based Approach

**DOI:** 10.3389/fmicb.2021.613697

**Published:** 2021-02-04

**Authors:** Fatemah Sadeghpour Heravi, Martha Zakrzewski, Hamidreza Aboulkheyr Estarabadi, Karen Vickery, Honghua Hu

**Affiliations:** ^1^Surgical Infection Research Group, Faculty of Medicine and Health Sciences, Macquarie University, Sydney, NSW, Australia; ^2^QIMR Berghofer Medical Research Institute, Brisbane, QLD, Australia; ^3^Faculty of Engineering and Information Technology, School of Biomedical Engineering, University of Technology Sydney, Sydney, NSW, Australia

**Keywords:** diabetic foot infections, RNA sequencing, chemokines, cytokines, immune system, wound healing 2

## Abstract

The normal continuity of skin tissue can be affected by invading pathogens and lead to a series of complicated physiological events. Using an RNA sequencing-based approach, we have captured a metatranscriptomic landscape from diabetic foot infections (DFIs). The hierarchical clustering of the top 2,000 genes showed the expression of four main clusters in DFIs (A, B, C, and D). Clusters A and D were enriched in genes mainly involved in the recruitment of inflammatory cells and immune responses and clusters B and C were enriched in genes related to skin cell development and wound healing processes such as extracellular structure organization and blood vessel development. Differential expression analysis showed more than 500 differentially expressed genes (DEGs) between samples with a low number of virulence factors and samples with a high number of virulence factors. Up-regulated and down-regulated genes were mainly involved in adaptive/native immune responses and transport of mature mRNAs, respectively. Our results demonstrated the importance of inflammatory cytokines of adaptive/native immunity in the progression of DFIs and provided a useful groundwork for capturing gene snapshots in DFIs. In addition, we have provided a general introduction to the challenges and opportunities of RNA sequencing technology in the evaluation of DFIs. Pathways identified in this study such as immune chemokines, Rho GTPases, and corresponding effectors might be important therapeutic targets in the management of DFIs.

## Introduction

Diabetic foot infections (DFIs) are the most severe and costly complication of diabetes developing in fifty percent of people with diabetic foot ulcers worldwide (Sadeghpour Heravi et al., 2019) and causing high mortality and disability (International Diabetes Federation, 2019). Sequential physiological events and several molecules and pathways controlled by wound-adjacent cells and affected cells are involved in DFIs development (Bekeschus et al., 2017).

Identification of physiological events and differentially expressed genes across conditions in DFIs have been limited to the hybridization-based microarray methods which can only profile predefined transcripts. Background hybridization noises and low sensitivity are other limitations in microarray studies (Rao et al., 2019).

Recently, RNA sequencing approach has emerged as an alternative technique for transcriptome-based applications beyond the limitations of hybridization-based microarray methods for gene expression profiling (Zhang et al., 2015). The recent advances in RNA sequencing technology not only saves cost and time but also enables transcriptional landscape in clinical samples.

The identification of genes, chemokines, and immune cells involved in DFIs is required for targeting the most relevant pathways (Rees et al., 2015). However, there is no information to provide a complete view of the host gene expression profile in DFIs. In this study, using an RNA sequencing-based method, we broadened our understanding of host inflammatory status that contributes to the development of DFIs.

It should be noted that with the quick growth of RNA sequencing application, considering an appropriate number of replicates is a critical step in experiment design. However, due to unpredicted technical challenges we encountered during conducting this study, the evaluation of DFIs gene expression was limited to a low number of sample size. Although this study laid the groundwork for the evaluation of gene expression analysis in DFIs, further investigation using a larger sample size is needed to verify the application and effectiveness of this method in DFIs management.

To the best of our knowledge, this is the first study to apply an RNA-sequencing analysis to evaluate host gene expression profiles in DFIs.

## Materials and Methods

### Patient Population

In this prospective study, forty-three consecutive patients with DFIs referring to the Liverpool Hospital High-Risk Foot Service were recruited over a 6-month period. Using the International Working Group of the Diabetic Foot (IWGDF), Perfusion, Extent, Depth, Infection, and Sensation (PEDIS) classification system, DFI severity was determined in patients (PEDIS 2: mild infection, PEDIS 3: moderate infection, PEDIS 4: severe infection) (Monteiro-Soares et al., 2020). Recruited patients did not receive any antimicrobial therapy 2 weeks prior to the sample collection.

### Sample Collection

**FIGURE 1:**
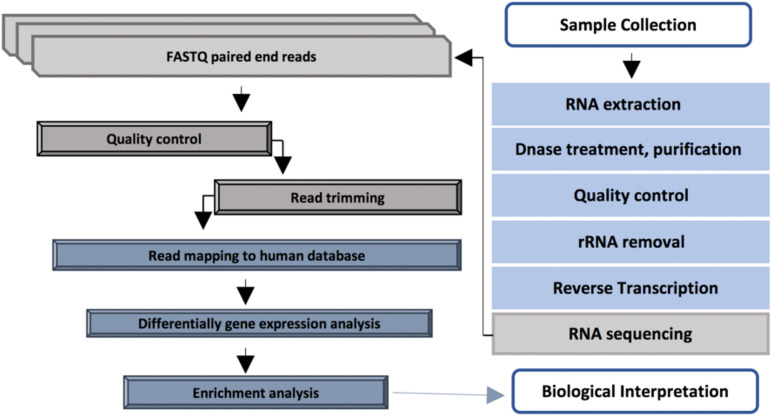
RNA sequencing pipeline applied in this study.

Diabetic foot infections were cleaned using sterile 0.9% NaCl and a sharp debridement was collected using a sterile single-use punch from the affected area. Collected tissue samples were preserved immediately in a 2 ml RNA*later* stabilization solution (Thermo Fisher Scientific, Waltham, MA, United States) for 24 h at 4°C and then stored at −80°C until extraction. The overview of the RNA analysis pipeline applied in this study is shown in Figure 1.

### Sample Preparation, RNA Extraction, and Illumina Sequencing

A detailed description of initial steps regarding sample preparation, RNA extraction, and sequencing process can be found in the previously described study (Heravi et al., 2020b).

### Processing of Host Transcripts

Read quality was assessed using Cutadapt v1.18 (Martin, 2011) by trimming adapters and removing flanking “N” bases in the paired-end sequences. The mapping of the trimmed sequencing was carried out with STAR v2.5.2a (Dobin et al., 2013) using the human GRCh37_ICGC_standard_v2 reference. Quality control was performed using RNA-SeQC v1.1.8 (DeLuca et al., 2012). To determine the expected read counts FPKM, RSEM v1.2.30 was used with a forward probability of 0 (Li and Dewey, 2011).

### Gene Ontology of Host Transcriptomes

To evaluate the potential biological role of the top 2,000 genes identified in the host, K-means was used to identify clusters of the highly expressed genes using iDEP.90 (9).

### Differentially Expressed Genes and Enrichment Analysis

Differentially expression analysis was performed between samples with a low number of virulence factors (control) and samples with a high number of virulence factors (perturbation) using BioJupies (Torre et al., 2018). The signature of gene expression was produced by comparing gene expression levels in the control and perturbation groups using the limma R package and visualized in a volcano plot (Ritchie et al., 2015). Functional analysis of identified genes was carried out in Enrichr (Chen et al., 2013).

## Results

Although RNA sequencing analysis is rapidly evolving, selecting the appropriate number of replicates has a significant impact on performance characteristics. However, due to unforeseen challenges including low RIN in extracted RNA samples, we had to select a few samples which met the metatranscriptomics requirements. After the initial assessment of extracted RNA, samples from sixteen patients presented with either a mild (25%), moderate (31.25%), or severe diabetic foot infection (43.75%) with high integrity and quality RNA required for RNA sequencing were selected. All patients suffered from peripheral neuropathy. No patient was present with immunosuppressive disease or cancer at the time of sample collection. Patients data can be found in Supplementary Table 1.

### Gene Ontology Analysis of Host Transcriptomes

**FIGURE 2:**
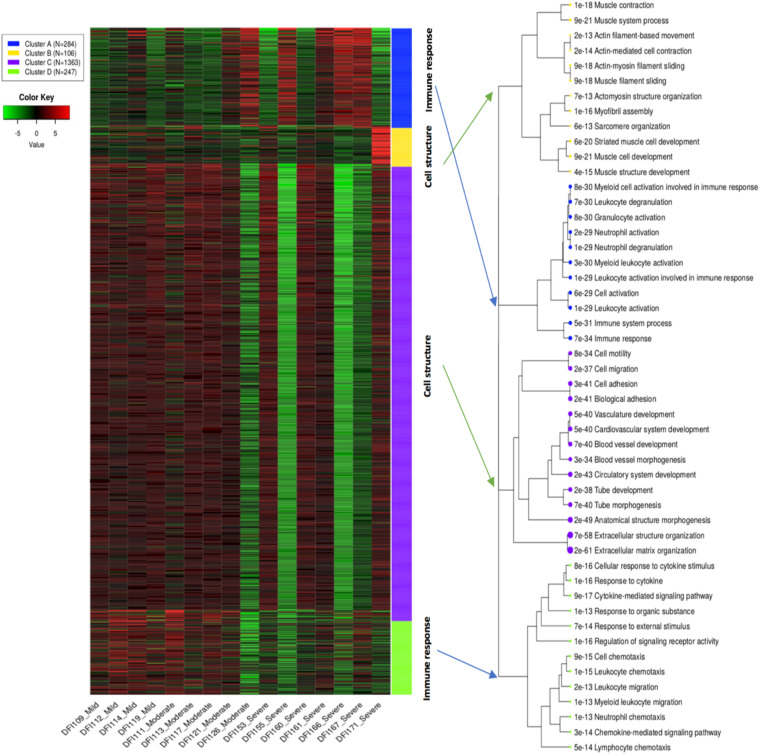
K-means clustering shows gene ontology enrichment analysis of the top 2,000 genes in DFIs. Four main clusters (A, B, C, and D) were associated with innate/adaptive immune responses, and biological functions in skin cells. The hierarchical tree indicates the relationship between gene ontology terms.

K-means clustering divided the top 2,000 genes into four main clusters including A, B, C, and D. Samples with mild and moderate infections had a relatively similar pattern compared to samples with a severe infection which may indicate similar expression pattern in the early stages of infection compared to late stages (Figure 2). Gene enrichment analysis indicated that cluster C as the biggest cluster (1,363 genes, *p* < 8.06E-34) and cluster B (106 genes, *p* < 6.76E-13) were strongly enriched in genes related to biological molecular mechanisms of skin cells such as extracellular structure organization, cell adhesion, and blood vessel development. Cluster A (284 genes, *p* < 6.13E-29) and cluster D (247 genes, *p* < 2.14E-13) comprised genes involved in immune and defense responses (Supplementary Table 2). Cluster D with high expression in mild/moderate samples and lower expression in severe samples showed the probable contribution of molecules and proteins leading to skin cell activities including enzyme production, cell movement, activation of cell receptors, and immune cell chemotaxis/migration. Likewise, Cluster C showed a similar pattern to cluster D regarding the mild/moderate and severe samples. This cluster with the probability of the presence of pathways involved in the development of anatomical structures of blood vessels and skin cells showed higher expression in mild/moderate and lower expression in severe samples. Clusters A and B showed variable patterns across the samples which were enriched with pathways involved in activation of granulocytes/immune cells and muscle cell development, respectively. However, it should be noted that enrichment analysis only reflects the detection of RNA transcripts and does not necessarily imply functional activity and the presence of related proteins.

### Differentially Expressed Genes (DEGs) and Enrichment Analysis

**FIGURE 3:**
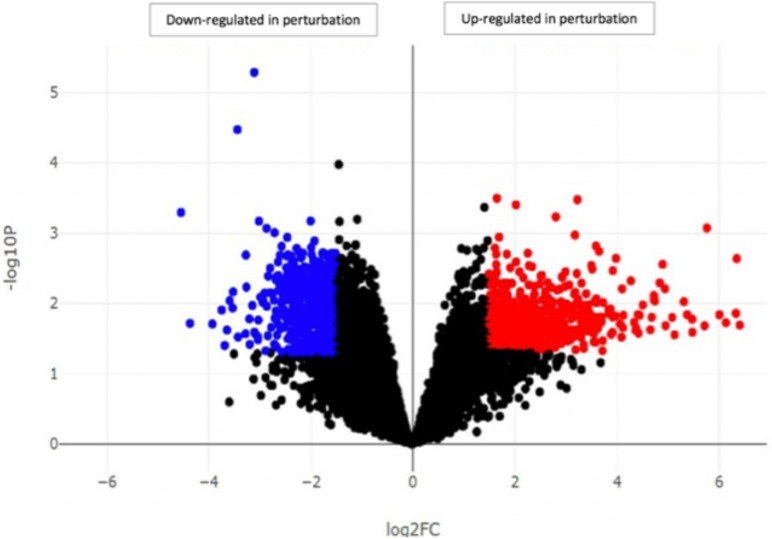
Identification of genes with significantly different expression in DFIs between samples with a high number of virulence factors (perturbation) and samples with a low number of virulence factors (control). Each point in the scatter plot shows a gene. The axes represent the significance vs. fold-change resulting from differential gene expression analysis. All the genes above the line log2FC *x* = 2 and below the line log2FC *x* = –2 were determined as up-regulated and down-regulated genes, respectively.

Our investigation identified the highest difference and the most interesting findings between samples with a low number of virulence factors (control) and samples with a high number of virulence factors (perturbation) which were defined previously (Heravi et al., 2020b). Differential gene expression analysis between control (DFI121, DFI109, DFI111, and DFI167) and perturbation groups (DFI166, DFI161, and DFI126) showed more than 500 differentially expressed genes (DEGs). Up-regulated and down-regulated genes were mainly involved in innate/adaptive immune systems and transport of mature mRNAs and histone mRNA, respectively (*p* < 0.05) (Figure 3).

**FIGURE 4:**
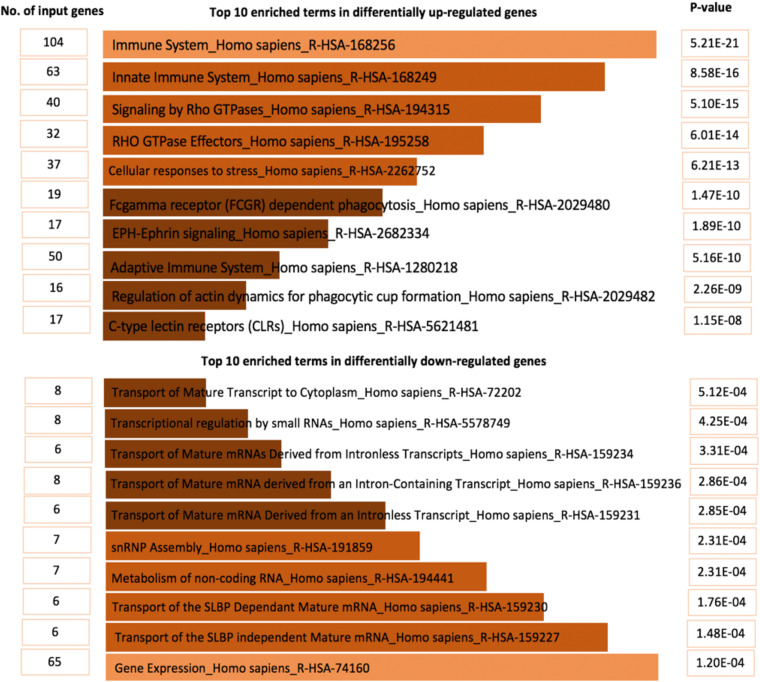
The top 10 enriched biological terms for DEGs.

The top ten enriched terms for up-regulated genes were mainly associated with the innate/adaptive immune systems, expression of Rho family protein, Fc gamma receptors (FCGRs), and re-arrangement of the actin cytoskeleton (*p* < 2.26E-09). Also, differentially down-regulated genes were mainly involved in transport of mature mRNA (*p* < 5.12E-04) (Figure 4 and Supplementary Tables 3, 4).

## Discussion

Diabetic foot infections are the most serious complication of diabetes and cause a high rate of morbidity, mortality, and healthcare costs (Heravi et al., 2020a). DFIs are the main cause of lower-extremity amputations and have debilitated many people with diabetes around the world (Vatankhah et al., 2017).

Although different adjuvant treatments have been introduced to manage DFIs, targeting essential molecular pathways involved in the development of DFIs is still a major challenge. By using hybridization methods (microarray), previous studies focused on the investigation of a limited number of chemokines and pathways involved in DFIs which may not reflect the complexity of gene expression profile and chemokine systems in DFIs. Also, in hybridization-based methods, prior knowledge of the gene sequence is a prerequisite that may not be possible in large scale studies. Background noises, cross-hybridization, and low sensitivity are other limitations in microarray studies (Nagalakshmi et al., 2008).

RNA sequencing has revolutionized our understanding of gene expression far beyond that of microarray studies. This technique is likely to become the frontline in transcriptome analysis as RNA sequencing pipelines continue to improve and costs continue to decrease. This approach has enabled the possibility of gene expression analysis of all involved genes at once in a complex condition and overcome limitations in hybridization-based methods (Bjornson et al., 2020).

Previous studies showed G proteins and chemotactic cytokines (C, CC, CXC, and CX3C) as major signaling molecules in the initiation and progression of DFIs (Pease and Williams, 2006; Vaseghi et al., 2017). Manipulation of signaling G proteins (Rho GTPases) and chemokine systems has led to many great achievements in the wound healing process (Satish, 2015). Also, recent studies have shown that over-expression of the CXC chemokine family (CXCL12) in wounds of diabetic mice has led to improved healing (Restivo et al., 2010).

However, these studies only focused on the expression of a limited number of genes and chemokines in DFIs, in the present study we took advantage of metatranscriptomic analysis and provided a comprehensive view of gene expressions in DFIs.

We have identified several genes encoding different chemokines and growth factors involved in the progression and wound healing process in DFIs in this study. The identified genes are mainly involved in the recruitment of immune and inflammatory cells and overlapping phases of wound healing (such as hemostasis, inflammation, proliferation, and maturation) and promotion of angiogenesis.

The overall expression profile of the top 2,000 genes in DFIs showed four main clusters (A, B, C, and D). Clusters A and D were enriched in genes involved in the immune system responses such as leukocyte activation, neutrophil degranulation, and chemotaxis/migration of leukocytes. Clusters B and C were enriched in genes related to molecular mechanisms in skin cells (such as the development of muscle cells, extracellular structure organization, and blood vessel development). Cluster D showed a high expression of genes involved in the migration of immune cells in early infections (mild/moderate) while cluster A showed high expression of genes associated with activation and degranulation of leukocyte in severe infections. However, it should be underlined that protein translation and functional activity can not necessarily be suggested by the existence of genes/transcripts.

Cluster D mainly contained genes coding the CXC chemokine family [e.g., CXCL6, CXCL9, and CXCL5 (neutrophil chemotaxis), CXCL3 (monocytes migration), and CXCL10 (macrophages, monocytes, NK cells, T cells, and dendritic cells chemotaxis)]. This cluster had a high expression in mild/moderate samples and lower expression in severe stages which may indicate a slow healing process in severe wounds. Low expression of the CXC chemokine family (CXCL6, CXCL9, CXCL5, CXCL3, and CXCL10) has also been previously associated with the development of non-healing wounds (Satish, 2015). Previous findings have also shown a predominance of CCR5, CCR3, CCR2, and CCR1 in early infections, while expression of CXCR1 and CXCR2 occurred in late infections possibly released by activated monocytes and other inflammatory cells (Baggiolini, 2001; Borish and Steinke, 2003). Clusters B and C with high expression in mild/moderate samples and low expression in severe samples were mainly comprised of genes involved in blood vessel morphogenesis and extracellular matrix organization (such as TIE1, 2, LMOD2, NEB, MYBPC1, ACTN2, and TNNC2). Although the progression of non-healing wounds is multifaceted, there is a strong correlation between the development of non-healing wounds and poor vascular network (Aronow and McClung, 2015). Among circulating signaling molecules and angiogenesis regulating factors, Tie1, 2 belonging to the angiopoietin family have a major role in vessel maturity (Sundberg et al., 2002). Based on previous findings high expression of microvesicular receptors Tie1, 2 have been associated with the normal repair while downregulation of angiogenesis regulating factors was associated with severe ulcers which were in accordance with our findings (Okonkwo and DiPietro, 2017).

Differentially expressed genes analysis between samples with low and high number virulence factors showed that up-regulated genes were mainly involved in innate/adaptive immune systems, while down-regulated genes were mainly associated with the transport of mature mRNAs/histone mRNA. The top 10 up-regulated terms were mainly involved in innate immune systems, Rho GTPases signaling pathway, Fc gamma receptor-dependent phagocytosis and adaptative immune systems (Figure 4) which indicated the significant role of inflammation mediated by chemokine and cytokine signaling pathway in DFIs development. High expression of genes involved in signaling G protein pathways, cytoskeletal function, and recruitment of inflammatory chemokines confirmed the necessity of immune cytokines and Rho family GTPases in the development of DFIs and were consistent with previous findings (Abreu-Blanco et al., 2014; Ridley, 2016).

Although RNA sequencing has played a significant role in transcriptome profiling, there are still limitations in this approach that requires to be addressed. When designing an RNA sequencing study, a few considerations should be taken into account. Budget management and the number of sample sizes have always been controversial in the RNA sequencing approach (Geraci et al., 2020). A larger sample size is contributed to increased statistical power and reduced undesired noises. Also, it should be noted that the extraction of high-quality RNA is a challenging process and requires careful precaution. Although after sampling, tissue samples had been immediately preserved in RNAlater solution in this study, after the initial assessment, we were limited to a few RNA samples which met the metatranscriptomics requirements. It is worth pointing out that all the precautions should be applied during the sample collection, storage, and RNA extraction procedure according to the sample type to minimize RNA degradation and improve the outcomes in metatranscriptomic studies.

## Conclusion

In this study, we evaluated host immune response in DFIs using an RNA sequencing-based approach. Identification of whole transcriptomic profiles using an RNA sequencing-based technology provided a molecular insight of host response in DFIs. Our results demonstrated the importance of inflammatory cytokines in adaptive/native immunity in the progression of DFIs. Pathways identified in this study particularly immune chemokines, Rho GTPases, and corresponding effectors might be important therapeutic targets through modulation of cytoskeletal function and manipulation of cytokines in the improvement of DFIs. However, further investigation in a larger scale is warranted to validate the findings in this study. Moreover, to investigate host-pathogen crosstalk in detail, pre-clinical studies using cell culture and animal models is required.

## Data Availability Statement

The datasets presented in this study can be found in online repositories. The names of the repository/repositories and accession number(s) can be found below: https://www.ncbi.nlm.nih.gov/, PRJNA563930.

## Ethics Statement

This study was approved by the South Western Sydney Local Health District Research and Ethics Committee (HREC/14/LPOOL/487, SSA/14/LPOOL/489) and Macquarie University Human Ethics Committee (Reference No. 5201500839). The patients/participants provided their written informed consent to participate in this study.

## Author Contributions

FH conducted the laboratory experiments and data analysis and drafted the manuscript. MZ and HA performed the data analysis and reviewed the manuscript. KV reviewed the manuscript. HH designed the project, monitored laboratory experiments, and reviewed the manuscript. All authors contributed to the article and approved the submitted version.

## Conflict of Interest

The authors declare that the research was conducted in the absence of any commercial or financial relationships that could be construed as a potential conflict of interest.
