# Supplementary material for: Evaluation of Host Immune Response in Diabetic Foot Infection Tissues Using an RNA Sequencing-Based Approach
Source: Front Microbiol. 2021 Feb 4;12:613697. doi: 10.3389/fmicb.2021.613697 (PMC7890089; doi:10.3389/fmicb.2021.613697)
Supplement: Supplementary file 1 [file Data_Sheet_1.docx]

**Supplementary**

Supplementary TABLE S1 Patients data investigated in this study

| Patient ID | PAD | CKD  stage 5 | CHD | CCF | WCC  (x10^9/L) | CRP  (mg/L) | ESR  (mL/min) | Neutrophils  (x10^9/L) | HBA1c (%) | PEDIS score  2= Mild, 3= Moderate, 4= Severe |
| --- | --- | --- | --- | --- | --- | --- | --- | --- | --- | --- |
| DFI109 |  |  |  |  | 9.8 | 48 | 54 | 7.1 | 8.1 | 2 |
| DFI111 |  |  |  |  | 11.1 | 28.9 | 40 | 8.1 | 8.2 | 3 |
| DFI112 |  |  |  |  | 7.1 | 13 | 25 | 5 | 7.5 | 2 |
| DFI113 |  |  | Yes |  | 9.7 | 17.8 | 34 | 6.2 | 12.6 | 3 |
| DFI114 |  |  | Yes |  | 9 | 17.4 | 62 | 11.9 | 10.7 | 2 |
| DFI117 |  |  |  |  | 8.8 | 26.9 | 128 | 6.3 | 13.6 | 3 |
| DFI119 |  |  |  |  | 6.3 | 0.6 | 22 | 3.1 | 6.2 | 2 |
| DFI121 |  |  |  |  | 14.7 | 83.8 | 80 | 12 | 7.3 | 3 |
| DFI126 | Yes | Yes |  |  | 18.5 | 187.4 | 119 | 16.2 | 17.3 | 3 |
| DFI153 | Yes |  |  | Yes | 9.3 | 21.5 |  | 6 | 9.4 | 4 |
| DFI155 | Yes |  |  |  | 15 | 386.2 |  | 13.1 | 9.8 | 4 |
| DFI160 |  |  |  |  | 12.4 | 46.4 | 66 | 8.8 | 9.8 | 4 |
| DFI161 |  |  |  |  | 12.5 | 55.8 |  | 9.2 | 11.4 | 4 |
| DFI166 | Yes |  | Yes |  | 10.8 | 38.4 | 52 | 8.8 | 8.2 | 4 |
| DFI167 |  |  |  | Yes | 12.5 | 35.6 | 82 | 9.4 | 8.7 | 4 |
| DFI171 | Yes |  |  |  | 11.4 | 32 |  | 7.8 | 11.6 | 4 |

*PAD: peripheral arterial disease, CKD: chronic kidney disease, CHD: coronary heart disease; CCF: congestive cardiac failure, WCC: White cell count, CRP:C-reactive protein, ESR: Erythrocyte sedimentation rate

Supplementary TABLE S2 Enrichment pathways for top 2000 genes in each cluster

| Cluster | adj. Pval | nGenes | Pathways | Genes |
| --- | --- | --- | --- | --- |
| A | 6.55E-34 | 89 | Immune response | CXCL2 MEFV TLR2 CXCR1 PTX3 PPBP CXCL1 CXCR2 C5AR1 DEFA1 DEFA3 CCL3 CCL4L2 CCL3L3 LTF LCP2 CEACAM1 TNFAIP3 SLPI APOBEC3A LCP1 ANXA3 NOD2 CD55 KRT6A PRKCQ MCOLN1 OSM NFKBIA CLEC4A VNN1 IL1A PTK2B TNFSF14 RAC2 ARRB2 ADAM8 GBP5 NLRP3 MNDA NFIL3 CLEC4E ISG20 CLEC7A KRT16 IL1RAP LST1 DEFB4A PDE4B HSPA1B HSPA1A MMP25 PLAUR ALOX5 SERPINB1 SERPINB3 SLC2A3 CST7 AQP9 ALDOC CD83 GCA IL1R2 NCF2 PADI2 CYSTM1 PI3 TREM1 GPR84 GPR65 ITGAX IGSF6 CD53 FCGR2A FCGR3B S100P FOS CEACAM3 LRG1 HSPA6 OLR1 SOCS3 FCAR SELL SERPINA1 ORM1 SIGLEC14 HP MGAM |
| A | 4.52E-31 | 98 | Immune system process | CXCL2 MEFV RAC2 TLR2 CXCR1 PTX3 PPBP CXCL1 CXCR2 C5AR1 DEFA1 DEFA3 CCL3 CCL4L2 CCL3L3 LTF LCP2 CEACAM1 CLEC4A PADI2 TNFAIP3 SLPI TNFSF14 APOBEC3A LCP1 ANXA3 PMAIP1 ADAM8 NOD2 DDIT4 HCAR2 CD55 LST1 KRT6A PRKCQ CST7 MCOLN1 OSM NFKBIA NAMPT RIPOR2 CD83 VNN1 IL1A CSF3R PTK2B ITGAX ARRB2 FCGR2A GBP5 NLRP3 MNDA NFIL3 CLEC4E RHOH FOS ISG20 CLEC7A OLR1 FAM20C PDE4B KRT16 SELL IL1RAP ORM1 HP DEFB4A EVI2B HSPA1B HSPA1A MMP25 PLAUR ALOX5 SERPINB1 SERPINB3 SLC2A3 AQP9 ALDOC GCA IL1R2 NCF2 CYSTM1 PI3 TREM1 GPR84 GPR65 IGSF6 CD53 FCGR3B S100P CEACAM3 LRG1 HSPA6 SOCS3 FCAR SERPINA1 SIGLEC14 MGAM |
| A | 2.67E-30 | 51 | Myeloid leukocyte activation | ANXA3 CXCR2 CCL3 LCP2 CST7 NAMPT RAC2 TLR2 RHOH C5AR1 MMP25 PLAUR LTF ALOX5 SERPINB1 SERPINB3 SLC2A3 CEACAM1 ALDOC VNN1 GCA PADI2 CYSTM1 SLPI GPR84 ITGAX CD53 FCGR2A ADAM8 FCGR3B CXCR1 MNDA PTX3 PPBP CXCL1 S100P CEACAM3 LRG1 HSPA6 OLR1 FCAR SELL CD55 SERPINA1 HSPA1B HSPA1A DEFA1 ORM1 SIGLEC14 HP MGAM |
| A | 7.03E-30 | 47 | Leukocyte degranulation | CEACAM1 ANXA3 CCL3 RAC2 MMP25 PLAUR LTF ALOX5 SERPINB1 SERPINB3 SLC2A3 ALDOC VNN1 GCA PADI2 CYSTM1 SLPI TLR2 GPR84 ITGAX CD53 FCGR2A ADAM8 FCGR3B CXCR1 MNDA PTX3 PPBP CXCL1 S100P CEACAM3 LRG1 HSPA6 OLR1 CXCR2 FCAR SELL CD55 SERPINA1 C5AR1 HSPA1B HSPA1A DEFA1 ORM1 SIGLEC14 HP MGAM |
| A | 8.26E-30 | 47 | Myeloid cell activation involved in immune response | ANXA3 CCL3 RAC2 MMP25 PLAUR LTF ALOX5 SERPINB1 SERPINB3 SLC2A3 CEACAM1 ALDOC VNN1 GCA PADI2 CYSTM1 SLPI TLR2 GPR84 ITGAX CD53 FCGR2A ADAM8 FCGR3B CXCR1 MNDA PTX3 PPBP CXCL1 S100P CEACAM3 LRG1 HSPA6 OLR1 CXCR2 FCAR SELL CD55 SERPINA1 C5AR1 HSPA1B HSPA1A DEFA1 ORM1 SIGLEC14 HP MGAM |
| A | 8.26E-30 | 46 | Granulocyte activation | ANXA3 CXCR2 CCL3 MMP25 PLAUR LTF ALOX5 SERPINB1 SERPINB3 SLC2A3 CEACAM1 ALDOC VNN1 GCA PADI2 CYSTM1 SLPI TLR2 GPR84 ITGAX CD53 FCGR2A ADAM8 FCGR3B CXCR1 MNDA PTX3 PPBP CXCL1 S100P CEACAM3 LRG1 HSPA6 OLR1 FCAR SELL CD55 SERPINA1 C5AR1 HSPA1B HSPA1A DEFA1 ORM1 SIGLEC14 HP MGAM |
| A | 1.01E-29 | 64 | Leukocyte activation | CEACAM1 CLEC4A TNFSF14 LCP1 ANXA3 NOD2 CXCR2 CD55 LST1 CCL3 LCP2 PRKCQ CST7 NAMPT CD83 VNN1 TNFAIP3 PTK2B RAC2 TLR2 ADAM8 NLRP3 CLEC4E RHOH C5AR1 RIPOR2 MNDA MMP25 PLAUR LTF ALOX5 SERPINB1 SERPINB3 SLC2A3 ALDOC GCA PADI2 CYSTM1 SLPI GPR84 ITGAX CD53 FCGR2A FCGR3B CXCR1 PTX3 PPBP CXCL1 S100P CEACAM3 LRG1 CLEC7A HSPA6 OLR1 FCAR SELL SERPINA1 HSPA1B HSPA1A DEFA1 ORM1 SIGLEC14 HP MGAM |
| A | 1.06E-29 | 45 | Neutrophil degranulation | ANXA3 MMP25 PLAUR LTF ALOX5 SERPINB1 SERPINB3 SLC2A3 CEACAM1 ALDOC VNN1 GCA PADI2 CYSTM1 SLPI TLR2 GPR84 ITGAX CD53 FCGR2A ADAM8 FCGR3B CXCR1 MNDA PTX3 PPBP CXCL1 S100P CEACAM3 LRG1 HSPA6 OLR1 CXCR2 FCAR SELL CD55 SERPINA1 C5AR1 HSPA1B HSPA1A DEFA1 ORM1 SIGLEC14 HP MGAM |
| A | 1.45E-29 | 51 | Leukocyte activation involved in immune response | CEACAM1 LCP1 ANXA3 CCL3 PTK2B RAC2 NLRP3 CLEC4E MMP25 PLAUR LTF ALOX5 SERPINB1 SERPINB3 SLC2A3 ALDOC VNN1 GCA PADI2 CYSTM1 SLPI TLR2 GPR84 ITGAX CD53 FCGR2A ADAM8 FCGR3B CXCR1 MNDA PTX3 PPBP CXCL1 S100P CEACAM3 LRG1 HSPA6 OLR1 CXCR2 FCAR SELL CD55 SERPINA1 C5AR1 HSPA1B HSPA1A DEFA1 ORM1 SIGLEC14 HP MGAM |
| A | 2.41E-29 | 45 | Neutrophil activation | ANXA3 CXCR2 MMP25 PLAUR LTF ALOX5 SERPINB1 SERPINB3 SLC2A3 CEACAM1 ALDOC VNN1 GCA PADI2 CYSTM1 SLPI TLR2 GPR84 ITGAX CD53 FCGR2A ADAM8 FCGR3B CXCR1 MNDA PTX3 PPBP CXCL1 S100P CEACAM3 LRG1 HSPA6 OLR1 FCAR SELL CD55 SERPINA1 C5AR1 HSPA1B HSPA1A DEFA1 ORM1 SIGLEC14 HP MGAM |
| A | 6.13E-29 | 66 | Cell activation | CEACAM1 CLEC4A TNFSF14 LCP1 TLR2 ANXA3 NOD2 CXCR2 CD55 LST1 CCL3 LCP2 PRKCQ CST7 NAMPT CD83 VNN1 TNFAIP3 PTK2B RAC2 ADAM8 NLRP3 CLEC4E RHOH C5AR1 RIPOR2 MNDA MMP25 PLAUR LTF ALOX5 SERPINB1 SERPINB3 SLC2A3 ALDOC GCA PADI2 CYSTM1 SLPI GPR84 ITGAX ARRB2 PIK3R5 CD53 FCGR2A FCGR3B CXCR1 PTX3 PPBP CXCL1 S100P CEACAM3 LRG1 CLEC7A HSPA6 OLR1 FCAR SELL SERPINA1 HSPA1B HSPA1A DEFA1 ORM1 SIGLEC14 HP MGAM |
| B | 8.87E-21 | 18 | Muscle cell development | MYF6 LMOD2 NEB MYBPC1 BIN1 ACTN2 CASQ2 LDB3 FLNC CASQ1 ACTA1 ACTC1 PI16 MYOZ1 STAC3 RYR1 MYH3 OBSCN |
| B | 8.87E-21 | 23 | Muscle system process | TNNC2 TNNC1 CASQ2 CASQ1 LMOD2 MYBPC1 MYH2 MYL1 ATP2A1 ACTN2 MYH1 BIN1 ACTA1 ACTC1 PI16 MYOZ1 RYR1 MYH3 MYH8 STAC3 SLN NEB TRDN |
| B | 6.31E-20 | 17 | Striated muscle cell development | MYF6 LMOD2 NEB MYBPC1 ACTN2 CASQ2 LDB3 FLNC CASQ1 ACTA1 ACTC1 PI16 MYOZ1 STAC3 RYR1 MYH3 OBSCN |
| B | 1.20E-18 | 20 | Muscle contraction | TNNC2 TNNC1 CASQ2 CASQ1 LMOD2 MYBPC1 MYH2 MYL1 ATP2A1 ACTN2 MYH1 BIN1 ACTC1 RYR1 MYH3 MYH8 STAC3 ACTA1 NEB TRDN |
| B | 9.24E-18 | 11 | Muscle filament sliding | TNNC1 MYH8 MYH2 MYL1 ACTN2 TNNC2 MYH3 ACTA1 ACTC1 NEB MYBPC1 |
| B | 9.24E-18 | 11 | Actin-myosin filament sliding | TNNC1 MYH8 ACTC1 MYH2 MYL1 ACTN2 TNNC2 MYH3 ACTA1 NEB MYBPC1 |
| B | 1.12E-16 | 12 | Myofibril assembly | LMOD2 NEB MYBPC1 CASQ2 LDB3 CASQ1 ACTC1 MYOZ1 ACTN2 ACTA1 MYH3 OBSCN |
| B | 3.65E-15 | 21 | Muscle structure development | MYF6 LMOD2 NEB MYBPC1 BIN1 ACTN2 CASQ2 LDB3 FLNC CASQ1 ACTA1 CXCL14 ACTC1 PI16 MYOZ1 STAC3 RYR1 TNNC1 RBP4 MYH3 OBSCN |
| B | 2.22E-14 | 12 | Actin-mediated cell contraction | TNNC1 BIN1 ACTC1 MYH8 MYH2 MYL1 ACTN2 TNNC2 MYH3 ACTA1 NEB MYBPC1 |
| B | 2.49E-13 | 12 | Actin filament-based movement | ACTC1 TNNC1 BIN1 MYH8 MYH3 MYH2 MYL1 ACTN2 TNNC2 ACTA1 NEB MYBPC1 |
| B | 6.48E-13 | 9 | Sarcomere organization | MYBPC1 CASQ2 LDB3 CASQ1 LMOD2 MYOZ1 ACTN2 MYH3 OBSCN |
| B | 6.76E-13 | 13 | Actomyosin structure organization | LMOD2 NEB MYBPC1 LIMCH1 CASQ2 LDB3 CASQ1 ACTC1 MYOZ1 ACTN2 ACTA1 MYH3 OBSCN |
| C | 2.48E-61 | 118 | Extracellular matrix organization | COL11A1 COL5A3 PLOD1 COL16A1 MMP2 MMP9 ECM2 COL1A1 TGFBI COL5A1 POSTN LOXL2 SULF1 MMP13 DPT COL8A1 SERPINH1 MMP14 DDR2 COL1A2 COL3A1 ANXA2 COL18A1 COL4A1 COL27A1 COL5A2 COL15A1 CST3 AEBP1 PXDN PDPN MFAP4 SMAD3 TPSAB1 ELN FBLN1 ADAMTS2 CCDC80 ABL1 COMP ENG COL12A1 SMOC2 LOX NID1 FMOD PRDX4 RAMP2 PDGFRA ETS1 EMILIN1 LUM FBLN5 SFRP2 CSGALNACT1 ITGB1 ABI3BP ACAN OLFML2B FBLN2 ADAMTS9 APBB2 EGFLAM PTK2 ANTXR1 LAMB2 EFEMP2 VWA1 GAS6 FLRT2 OLFML2A EGFL6 PHLDB2 GPM6B LAMC1 CLASP2 SH3PXD2B COL14A1 ITGA3 DCN TIMP2 VCAN TNC LAMA3 ITGB5 NID2 PDGFB CTSG SH3PXD2A VWF LAMA4 SPARC COL7A1 MFAP2 LRP1 KDR LOXL1 LAMA5 ITGB4 ITGA7 THBS1 ITGA11 COL6A1 HSPG2 CTSK ADAM12 JAM2 ADAMTS5 ADAMTS4 VCAM1 CAPN2 HTRA1 JAM3 A2M BGN LAMA2 MFAP5 ITGA1 |
| C | 6.86E-58 | 123 | Extracellular structure organization | COL11A1 COL5A3 PLOD1 COL16A1 MMP2 MMP9 ECM2 COL1A1 TGFBI APOE COL5A1 POSTN LOXL2 SULF1 MMP13 DPT COL8A1 SERPINH1 MMP14 DDR2 COL1A2 COL3A1 ANXA2 COL18A1 COL4A1 COL27A1 COL5A2 COL15A1 CST3 PLTP AEBP1 APOC1 PXDN PDPN MFAP4 SMAD3 TPSAB1 CD34 ELN FBLN1 ADAMTS2 CCDC80 ABL1 COMP ENG COL12A1 SMOC2 LOX NID1 FMOD PRDX4 RAMP2 PDGFRA ETS1 EMILIN1 LUM FBLN5 SFRP2 CSGALNACT1 ITGB1 ABI3BP ACAN OLFML2B FBLN2 ADAMTS9 APBB2 EGFLAM PTK2 ANTXR1 LAMB2 EFEMP2 VWA1 GAS6 FLRT2 OLFML2A EGFL6 PHLDB2 GPM6B LAMC1 CLASP2 SH3PXD2B COL14A1 ITGA3 DCN TIMP2 VCAN TNC LAMA3 ITGB5 NID2 PDGFB CTSG SH3PXD2A VWF LAMA4 SPARC COL7A1 MFAP2 LRP1 KDR LOXL1 LAMA5 ITGB4 ITGA7 THBS1 ITGA11 COL6A1 HSPG2 PRKACB CTSK ADAM12 JAM2 ADAMTS5 ADAMTS4 VCAM1 CAPN2 HTRA1 JAM3 A2M BGN LAMA2 MFAP5 ITGA1 |
| C | 2.13E-49 | 302 | Anatomical structure morphogenesis | PLXND1 CD9 RUFY3 EPHA3 TIE1 NEDD4 LRP6 RHOBTB1 COL12A1 PTK7 SEMA5A AMOTL2 COL7A1 PLXNA1 TEK RAMP3 OBSL1 COL21A1 RHOJ KDR PALLD RAMP2 MAP1B MATN2 SULF1 MMP13 THBS1 CDH11 CDH13 COL6A1 THY1 ROBO4 RHOC AUTS2 NEXN LMOD1 PLXNB1 DACT1 AMOTL1 SCARA3 PTK2 SDC2 CLIC4 MMRN2 FZD4 VWA1 ANXA2 THBS2 COL14A1 EPHB4 PLXNB2 ANXA6 PARVA ECSCR CYFIP1 ELN DCN HGF TGFBR3 VASH1 FBLN1 ASPN SMOC2 SPARC CLU PLCG1 COL5A1 LAMA5 LAMC1 FGF7 TAOK2 ADAMTS12 ANGPT1 NOS3 JAM3 ANTXR1 ROBO1 CD34 SLIT3 COL5A2 FYN WWTR1 SNAI2 EHD2 TNC COL11A1 NTN1 HYAL2 RORA FERMT2 NTN4 DLG1 FGFR1 RAPGEF3 ATP8B1 MMP2 DPYSL2 HDAC6 ABL1 NRP1 PALMD RBFOX2 NFATC4 SRPX2 TJP1 IMPAD1 SFRP1 PTPRS COMP SSBP1 ENG CXCL12 PLEKHA1 BMPR1A LIPA LZTS2 COL1A1 UGDH ELK3 MDFI LOX TANC1 PRRX1 STMN1 NRP2 PKD2 TGFB3 LRP1 RUNX2 PAX8 ID1 BCL11B PTPRB APOE NES MYH10 DCLK1 POSTN PTGFRN APLNR PDGFRA ETS1 ITGA7 FLNB ENPP2 TRPC6 YAP1 EMILIN1 MYOF ENPEP LRIG3 ACVRL1 FRMD6 TPM1 MFGE8 SS18 TP53 HSPG2 PRKACB CRABP2 COL8A1 BOC SFRP2 GPC3 CSGALNACT1 NFIB SERPINH1 ITGB1 EXT2 EPS8 EDNRA PTPN14 GJA1 FARP1 DDAH1 ADAMTS1 ADAMTS5 CD109 MMP14 FZD1 PALM2-AKAP2 ACAN NBL1 KALRN PLXDC1 EMC10 FMNL3 FBLIM1 PDPN KIF26B CAPN2 POGLUT1 ADAMTS9 APBB2 SPRY1 FZD6 CTHRC1 CFL2 HTRA1 SPRED1 DCHS1 WEE1 SMAD3 AXL COL3A1 ROR2 THBS3 TM4SF1 SMAD1 SGCD S1PR1 INSR APLN LAMB2 ID4 CYP7B1 EGFL7 RAPH1 HEG1 SH3PXD2B CD248 CLEC14A RCC2 MAGED1 CSF1R COL18A1 GJC1 GPC6 JAG2 FLRT2 PBX1 DYNC2H1 COL4A1 FAT4 LAMA2 TCF4 COL27A1 LPAR1 COL15A1 TCTN1 LGR4 ITGA1 SHANK3 GJA5 ITGA3 LAMA3 ITGB5 MMP9 CST3 EFEMP1 MFAP2 TGFBI ITGB4 EMCN AQP1 STARD13 PHLDB2 JUP STAB1 MYLK HSPB1 SH3PXD2A SH3D19 C12orf57 SASH1 RAB23 PTGIS LOXL2 RDX PRICKLE1 TBCD ADAM12 DOCK1 CACNA1C DAB2 CLASP2 COL1A2 DLC1 INF2 TSC2 ACTA2 AHI1 FAM129B FGD2 PTPRD MOV10 MTSS1 FHL1 DAAM1 CXCL13 PRICKLE2 EVL DKK3 FAT1 ABLIM1 LZTR1 TMEM176B MGP STX2 LYVE1 BICD1 F2R |
| C | 1.51E-43 | 166 | Circulatory system development | MYLK TIE1 LRP6 COL1A1 AMOTL2 TEK RAMP3 OBSL1 KDR RAMP2 APLNR SULF1 THBS1 CDH13 THY1 AMOTL1 PTK2 CLIC4 SGCD MMRN2 HEG1 FZD4 ANXA2 THBS2 ECSCR ELN BMPR1A DCN IGF1 TGFBR3 VASH1 PDGFB SMOC2 SPARC PLCG1 PRICKLE1 ANGPT1 NOS3 JAM3 CD34 EPHB4 PLXND1 ITGA3 SNAI2 HGF ADAMTS6 COL11A1 NEDD4 FGFR1 RAPGEF3 CAD MMP2 ABL1 NRP1 NFATC4 SRPX2 COMP ENG ELK3 PTK7 LAMA4 LOX PRRX1 NRP2 PKD2 KCNJ8 BICC1 PAX8 ID1 RHOJ PTPRB LOXL1 APOE COL5A1 MYH10 CCNB1 PDGFRA ETS1 ENPP2 YAP1 EMILIN1 ENPEP ACVRL1 TPM1 MFGE8 TP53 HSPG2 COL8A1 SFRP2 GPC3 ITGB1 EDNRA PTPN14 GJA1 DDAH1 ROBO4 PDLIM3 ADAMTS1 MMP14 FZD1 ACAN LMNA PLXDC1 EMC10 FMNL3 VCAM1 SGCB POGLUT1 ADAMTS9 SPRY1 CASP7 SPRED1 DCHS1 SMAD3 COL3A1 ANTXR1 ROBO1 CDK1 SMAD1 S1PR1 APLN EGFL7 SH3PXD2B CLEC14A COL18A1 GJC1 SLIT3 FLRT2 DYNC2H1 COL4A1 GJA4 FAT4 TCF4 PARVA PLN COL15A1 GJA5 ACTA2 TGFBI EMCN STARD13 JUP STAB1 RORA HSPB1 SASH1 PTGIS LOXL2 ADAM12 CACNA1C COL1A2 INSR AQP1 HECTD1 TSC2 SFRP1 SEMA5A WDR11 LRP1 AHI1 FAM129B SORBS2 PDPN DLC1 LAMA5 CXCL13 |
| C | 1.94E-41 | 200 | Biological adhesion | PTPRS PTPRD MYL9 HSPB1 PLXND1 CD9 DLG1 COL5A3 ITGB5 COL16A1 ECM2 PLXNA1 PCDH17 TGFBI PALLD COL5A1 ITGB4 POSTN EMILIN1 MMRN1 CDH11 CDH13 TINAGL1 HMCN1 ITGB1 THY1 ROBO4 PCDH1 NEXN VCAM1 DDR2 FBLN2 PLXNB1 NLGN4Y PTK2 MMRN2 PCDH18 PLXNB2 PARVA ITGBL1 SPON1 SNAI2 SELE IGF1 EPHA3 FBLN1 CD209 NRP1 SRPX2 CD276 CXCL12 VWF KDR LAMA5 AOC3 LAMC1 CCL21 THBS1 ADAMTS18 TAOK2 SPOCK1 ANGPT1 PDPN IGFBP7 EMCN DLC1 COL3A1 ANTXR1 ROBO1 JUP CD34 SELP RCC2 GAS6 EPHB4 CD99 ITGA3 STAB1 VCAN TNC KITLG BCAR1 LAMA3 NTN1 LRP6 FERMT2 FAT1 NID2 CCDC80 SORBS1 ABL1 LGALS1 COMP ENG MPDZ PDLIM1 LGALS3BP COL1A1 COL12A1 SMOC2 PTK7 LAMA4 COL7A1 NID1 CNN3 LRP1 ID1 ZBTB1 OMD MYH10 LYVE1 ITGA7 ITGA11 PDE5A FBLN5 HAPLN3 MFGE8 COL6A1 DPT COL8A1 BOC ADAM12 ESAM DST SPARCL1 PTPRK ABI3BP JAM2 VSIG4 MMP14 ACAN FBLIM1 MXRA8 KIF26B ADAMTS9 EDIL3 EGFLAM SVEP1 JAM3 DCHS1 MFAP4 SMAD3 AXL PRDX2 RPSA THBS3 S1PR1 SOCS5 CLSTN1 LAMB2 NEGR1 EGFL7 FZD4 ANXA2 COL18A1 JAG2 FLRT2 THBS2 BCAM COL14A1 FAT4 LAMA2 EGFL6 COL15A1 ITGA1 GPM6B PIEZO1 TEK PDGFRA ETS1 RDX ACVRL1 PHLDB2 DOCK1 ADAMTS12 CLASP2 HLA-DPA1 LRRC15 HLA-DPB1 HLA-DMB SFRP1 SEMA5A TPM1 TBCD SFRP2 CXCL13 HLA-DRB1 ARL2 PPP2R1A NRP2 TPBG MTSS1 FYN PPFIBP1 SSPN SGCE ISLR LOXL2 ATP2A2 NME2 |
| C | 3.00E-41 | 199 | Cell adhesion | PTPRS PTPRD MYL9 HSPB1 PLXND1 CD9 DLG1 COL5A3 ITGB5 COL16A1 ECM2 PLXNA1 PCDH17 TGFBI PALLD COL5A1 ITGB4 POSTN EMILIN1 MMRN1 CDH11 CDH13 TINAGL1 HMCN1 ITGB1 THY1 ROBO4 PCDH1 NEXN VCAM1 DDR2 FBLN2 PLXNB1 NLGN4Y PTK2 MMRN2 PCDH18 PLXNB2 PARVA ITGBL1 SPON1 SNAI2 SELE IGF1 EPHA3 FBLN1 CD209 NRP1 SRPX2 CD276 CXCL12 VWF KDR LAMA5 AOC3 LAMC1 CCL21 THBS1 ADAMTS18 TAOK2 SPOCK1 ANGPT1 PDPN IGFBP7 EMCN DLC1 COL3A1 ANTXR1 ROBO1 JUP CD34 SELP RCC2 EPHB4 CD99 ITGA3 STAB1 VCAN TNC KITLG BCAR1 LAMA3 NTN1 LRP6 FERMT2 FAT1 NID2 CCDC80 SORBS1 ABL1 LGALS1 COMP ENG MPDZ PDLIM1 LGALS3BP COL1A1 COL12A1 SMOC2 PTK7 LAMA4 COL7A1 NID1 CNN3 LRP1 ID1 ZBTB1 OMD MYH10 LYVE1 ITGA7 ITGA11 PDE5A FBLN5 HAPLN3 MFGE8 COL6A1 DPT COL8A1 BOC ADAM12 ESAM DST SPARCL1 PTPRK ABI3BP JAM2 VSIG4 MMP14 ACAN FBLIM1 MXRA8 KIF26B ADAMTS9 EDIL3 EGFLAM SVEP1 JAM3 DCHS1 MFAP4 SMAD3 AXL PRDX2 RPSA THBS3 S1PR1 SOCS5 CLSTN1 LAMB2 NEGR1 EGFL7 FZD4 ANXA2 COL18A1 GAS6 JAG2 FLRT2 THBS2 BCAM COL14A1 FAT4 LAMA2 EGFL6 COL15A1 ITGA1 GPM6B PIEZO1 TEK PDGFRA ETS1 RDX ACVRL1 PHLDB2 DOCK1 ADAMTS12 CLASP2 HLA-DPA1 HLA-DPB1 HLA-DMB SFRP1 SEMA5A TPM1 TBCD SFRP2 CXCL13 HLA-DRB1 ARL2 PPP2R1A NRP2 TPBG MTSS1 FYN PPFIBP1 SSPN SGCE ISLR LOXL2 ATP2A2 NME2 |
| C | 4.76E-40 | 129 | Cardiovascular system development | MYLK TIE1 COL1A1 AMOTL2 TEK RAMP3 KDR RAMP2 APLNR SULF1 THBS1 CDH13 THY1 AMOTL1 PTK2 CLIC4 MMRN2 FZD4 ANXA2 THBS2 ECSCR DCN VASH1 PDGFB SMOC2 SPARC PLCG1 ANGPT1 NOS3 JAM3 CD34 EPHB4 PLXND1 HGF ADAMTS6 NEDD4 FGFR1 RAPGEF3 MMP2 ABL1 NRP1 NFATC4 SRPX2 COMP ENG BMPR1A ELK3 PTK7 LAMA4 LOX PRRX1 NRP2 PKD2 ID1 RHOJ PTPRB LOXL1 APOE COL5A1 MYH10 PDGFRA ETS1 ENPP2 YAP1 EMILIN1 ENPEP PRICKLE1 ACVRL1 MFGE8 HSPG2 COL8A1 SFRP2 GPC3 ITGB1 EDNRA PTPN14 GJA1 DDAH1 ROBO4 MMP14 PLXDC1 EMC10 FMNL3 SGCB POGLUT1 ADAMTS9 SPRED1 COL3A1 ANTXR1 SGCD S1PR1 APLN EGFL7 HEG1 CLEC14A COL18A1 GJC1 DYNC2H1 COL4A1 GJA4 TCF4 PARVA COL15A1 GJA5 ACTA2 TGFBI EMCN STARD13 JUP STAB1 RORA HSPB1 SASH1 PTGIS LOXL2 ADAM12 COL1A2 ROBO1 SMAD1 AQP1 TGFBR3 HECTD1 SFRP1 SEMA5A LRP1 FAM129B PDPN LAMA5 CXCL13 |
| C | 5.33E-40 | 128 | Vasculature development | TIE1 COL1A1 AMOTL2 TEK RAMP3 KDR RAMP2 APLNR SULF1 THBS1 CDH13 THY1 AMOTL1 PTK2 CLIC4 MMRN2 FZD4 ANXA2 THBS2 ECSCR DCN VASH1 PDGFB SMOC2 SPARC PLCG1 ANGPT1 NOS3 JAM3 CD34 EPHB4 PLXND1 HGF ADAMTS6 NEDD4 FGFR1 RAPGEF3 MMP2 ABL1 NRP1 NFATC4 SRPX2 COMP ENG BMPR1A ELK3 PTK7 LAMA4 LOX PRRX1 NRP2 PKD2 ID1 RHOJ PTPRB LOXL1 APOE COL5A1 MYH10 PDGFRA ETS1 ENPP2 YAP1 EMILIN1 ENPEP PRICKLE1 ACVRL1 MFGE8 HSPG2 COL8A1 SFRP2 GPC3 ITGB1 EDNRA PTPN14 GJA1 DDAH1 ROBO4 MMP14 PLXDC1 EMC10 FMNL3 SGCB ADAMTS9 SPRED1 COL3A1 ANTXR1 SGCD S1PR1 APLN EGFL7 HEG1 CLEC14A COL18A1 GJC1 DYNC2H1 COL4A1 GJA4 TCF4 PARVA COL15A1 GJA5 ACTA2 TGFBI EMCN STARD13 JUP STAB1 MYLK RORA HSPB1 SASH1 PTGIS LOXL2 ADAM12 COL1A2 ROBO1 SMAD1 AQP1 TGFBR3 HECTD1 SFRP1 SEMA5A LRP1 FAM129B PDPN LAMA5 CXCL13 |
| C | 7.44E-40 | 125 | Blood vessel development | TIE1 COL1A1 AMOTL2 TEK RAMP3 KDR RAMP2 APLNR SULF1 THBS1 CDH13 THY1 AMOTL1 PTK2 CLIC4 MMRN2 ANXA2 THBS2 ECSCR DCN VASH1 PDGFB SMOC2 SPARC PLCG1 ANGPT1 NOS3 JAM3 CD34 EPHB4 PLXND1 HGF ADAMTS6 NEDD4 FGFR1 RAPGEF3 MMP2 ABL1 NRP1 NFATC4 SRPX2 COMP ENG BMPR1A ELK3 PTK7 LAMA4 LOX PRRX1 NRP2 PKD2 ID1 RHOJ PTPRB LOXL1 APOE COL5A1 MYH10 PDGFRA ETS1 ENPP2 YAP1 EMILIN1 ENPEP PRICKLE1 ACVRL1 MFGE8 HSPG2 COL8A1 SFRP2 GPC3 ITGB1 EDNRA GJA1 DDAH1 ROBO4 MMP14 PLXDC1 EMC10 FMNL3 ADAMTS9 SPRED1 COL3A1 ANTXR1 SGCD S1PR1 APLN EGFL7 HEG1 FZD4 CLEC14A COL18A1 GJC1 DYNC2H1 COL4A1 GJA4 TCF4 PARVA COL15A1 GJA5 ACTA2 TGFBI EMCN STARD13 JUP STAB1 MYLK RORA HSPB1 SASH1 PTGIS LOXL2 ADAM12 COL1A2 ROBO1 SMAD1 AQP1 TGFBR3 HECTD1 SFRP1 SEMA5A LRP1 FAM129B LAMA5 CXCL13 |
| C | 7.44E-40 | 141 | Tube morphogenesis | TIE1 LRP6 AMOTL2 TEK RAMP3 KDR RAMP2 SULF1 THBS1 CDH13 THY1 AMOTL1 PTK2 CLIC4 MMRN2 ANXA2 THBS2 ECSCR DCN VASH1 SMOC2 SPARC PLCG1 ADAMTS12 ANGPT1 NOS3 JAM3 CD34 EPHB4 PLXND1 HGF TNC NTN1 NEDD4 DLG1 FGFR1 RAPGEF3 MMP2 ABL1 NRP1 NFATC4 SRPX2 SFRP1 COMP ENG BMPR1A LZTS2 ELK3 PTK7 LOX PRRX1 NRP2 PKD2 PAX8 ID1 RHOJ PTPRB APOE LAMA5 APLNR PDGFRA ETS1 ENPP2 YAP1 EMILIN1 ENPEP ACVRL1 MFGE8 HSPG2 PRKACB COL8A1 SFRP2 GPC3 NFIB ITGB1 EDNRA GJA1 DDAH1 ROBO4 MMP14 FZD1 PLXDC1 EMC10 FMNL3 KIF26B ADAMTS9 SPRY1 FZD6 CTHRC1 SPRED1 DCHS1 SMAD3 COL3A1 SGCD S1PR1 APLN EGFL7 HEG1 FZD4 CLEC14A MAGED1 COL18A1 GJC1 PBX1 COL4A1 FAT4 PLXNB2 TCF4 PARVA COL15A1 TCTN1 LGR4 SHANK3 GJA5 TGFBI EMCN STARD13 JUP STAB1 MYLK RORA HSPB1 SASH1 PTGIS LOXL2 PRICKLE1 ADAM12 ROBO1 SMAD1 AQP1 TGFBR3 TSC2 SEMA5A LRP1 AHI1 FAM129B DLC1 DACT1 MTSS1 CXCL13 CSF1R |
| C | 2.04E-38 | 156 | Tube development | TIE1 LRP6 AMOTL2 TEK RAMP3 KDR RAMP2 SULF1 THBS1 CDH13 THY1 AMOTL1 PTK2 CLIC4 MMRN2 ANXA2 THBS2 ECSCR DCN VASH1 SMOC2 SPARC PLCG1 FGF7 ADAMTS12 ANGPT1 NOS3 JAM3 CD34 EPHB4 PLXND1 WWTR1 HGF TNC NTN1 NEDD4 DLG1 FGFR1 RAPGEF3 AKR1B1 ADAMTS2 MMP2 ABL1 NRP1 NFATC4 SRPX2 SFRP1 COMP PHF14 ENG BMPR1A LIPA LZTS2 ELK3 PTK7 LOX PRRX1 NRP2 PKD2 TGFB3 PAX8 ID1 RHOJ PTPRB APOE LAMA5 CCNB1 APLNR PDGFRA ETS1 TNS3 ENPP2 YAP1 EMILIN1 ENPEP ACVRL1 MFGE8 HSPG2 PRKACB COL8A1 SFRP2 GPC3 NFIB ITGB1 EDNRA GJA1 DDAH1 ROBO4 MMP14 FZD1 PLXDC1 EMC10 FMNL3 NFIA KIF26B ADAMTS9 SPRY1 FZD6 CTHRC1 SPRED1 DCHS1 SMAD3 COL3A1 SMAD1 SGCD S1PR1 APLN EGFL7 HEG1 FZD4 CLEC14A MAGED1 COL18A1 GJC1 PBX1 COL4A1 FAT4 PLXNB2 TCF4 PARVA COL15A1 TCTN1 LGR4 SHANK3 GJA5 TGFBI EMCN STARD13 JUP ITGA3 STAB1 MYLK RORA HSPB1 RARRES2 SASH1 PTGIS ITGB4 LOXL2 PRICKLE1 ADAM12 DACT1 CLMP ROBO1 AQP1 TGFBR3 TSC2 SEMA5A LRP1 AHI1 FAM129B PDPN DLC1 MTSS1 CXCL13 CSF1R |
| C | 2.05E-37 | 189 | Cell migration | PLXND1 HGF BCAR1 RHOBTB1 ITGB5 PDGFB CXCL12 SEMA5A AMOTL2 PLXNA1 TEK PLCG1 KDR MTUS1 ITGB4 PIK3C2B NAV1 CCL21 ITGB1 THY1 RHOC CXCL13 AUTS2 PLXNB1 AMOTL1 AXL SDC2 MMRN2 CMKLR1 CD248 CLEC14A PDXDC1 PLXNB2 ANXA6 ITGBL1 CCL18 CCL14 DCN IGF1 RUFY3 MYLK HYAL2 VASH1 FGFR1 LGMN SFRP1 ENG COL1A1 SASH1 SMOC2 SPARC APOE LAMA5 PDGFRA ENPP2 THBS1 ENPEP ACVRL1 FGF7 CDH13 IGFBP3 LRP12 PTPRK ANGPT1 MMP14 NEXN VCAM1 DLC1 NOS3 JAM3 ROR2 PTK2 CLIC4 ROBO1 PODN GAS6 GPC6 EPHB4 ITGA3 CD9 FYN SNAI2 FUT8 KITLG LIMA1 LAMA3 NTN1 TIE1 GSTP1 ASAP3 ABL1 NRP1 RBFOX2 MMP9 SRPX2 MDK LAMA4 LOX DPYSL3 NRP2 LRP1 NUP85 PALLD MATN2 MYH10 DCLK1 POSTN ETS1 TNS3 IL33 EMILIN1 SFRP2 DOCK1 DIXDC1 EPS8 GJA1 NBL1 LMNA FMNL3 DDR2 ADAMTS9 APBB2 CTHRC1 SPRED1 DCHS1 SMAD3 COL3A1 LDB2 S1PR1 CYP7B1 CD34 SELP ANO6 RCC2 F2R CSF1R COL18A1 FLRT2 LAMA2 KANK2 PARVA LPAR1 ITGA1 STARD13 ADAMTS1 JUP SELE SLC12A2 HDAC6 HSPB1 RARRES2 BMPR1A PTK7 RHOJ BCL11B PLVAP COL5A1 LOXL2 LAMC1 SULF1 RDX ITGA11 TPM1 CDC42BPA PTPRG PHLDB2 ADAMTS12 DAB2 PDPN CLASP2 INSR MCC LRRC15 EVL EPHA3 TGFBR3 FAT1 GPC3 SPOCK1 EMC10 TAOK2 ROBO4 ID1 MAP1B ESAM JAM2 COL1A2 CDK1 PROS1 |
| C | 3.11E-34 | 109 | Blood vessel morphogenesis | TIE1 AMOTL2 TEK RAMP3 KDR RAMP2 SULF1 THBS1 CDH13 THY1 AMOTL1 PTK2 CLIC4 MMRN2 ANXA2 THBS2 ECSCR DCN VASH1 SMOC2 SPARC PLCG1 ANGPT1 NOS3 JAM3 CD34 EPHB4 PLXND1 HGF NEDD4 FGFR1 RAPGEF3 MMP2 ABL1 NRP1 NFATC4 SRPX2 COMP ENG BMPR1A ELK3 LOX PRRX1 NRP2 ID1 RHOJ PTPRB APOE APLNR PDGFRA ETS1 ENPP2 YAP1 EMILIN1 ENPEP ACVRL1 MFGE8 HSPG2 COL8A1 SFRP2 ITGB1 EDNRA GJA1 DDAH1 ROBO4 MMP14 PLXDC1 EMC10 FMNL3 ADAMTS9 SPRED1 COL3A1 SGCD S1PR1 APLN EGFL7 HEG1 FZD4 CLEC14A COL18A1 GJC1 COL4A1 TCF4 PARVA COL15A1 GJA5 PKD2 TGFBI EMCN STARD13 JUP STAB1 MYLK RORA HSPB1 SASH1 PTGIS LOXL2 ADAM12 ROBO1 SMAD1 AQP1 TGFBR3 SFRP1 SEMA5A LRP1 FAM129B LAMA5 CXCL13 |
| C | 8.06E-34 | 194 | Cell motility | PLXND1 HGF BCAR1 RHOBTB1 ITGB5 PDGFB CXCL12 SEMA5A AMOTL2 PLXNA1 TEK PLCG1 KDR MTUS1 ITGB4 PIK3C2B NAV1 CCL21 ITGB1 THY1 RHOC CXCL13 AUTS2 PLXNB1 AMOTL1 AXL SDC2 MMRN2 CMKLR1 CD248 CLEC14A PDXDC1 PLXNB2 ANXA6 ITGBL1 CCL18 CCL14 DCN IGF1 RUFY3 MYLK HYAL2 VASH1 FGFR1 FBLN1 LGMN SRPX2 SFRP1 ENG COL1A1 SASH1 SMOC2 SPARC APOE LAMA5 PDGFRA ENPP2 THBS1 ENPEP ACVRL1 FGF7 CDH13 IGFBP3 LRP12 PTPRK ANGPT1 MMP14 NEXN VCAM1 DLC1 NOS3 JAM3 ROR2 PTK2 CLIC4 ROBO1 PODN GAS6 GPC6 EPHB4 ITGA3 CD9 FYN SNAI2 FUT8 KITLG LIMA1 LAMA3 NTN1 TIE1 GSTP1 ASAP3 ABL1 NRP1 RBFOX2 PLTP MMP9 MDK LAMA4 LOX DPYSL3 NRP2 LRP1 NUP85 PALLD MATN2 MYH10 DCLK1 POSTN ETS1 TNS3 IL33 ADCY3 EMILIN1 SFRP2 DOCK1 DIXDC1 EPS8 GJA1 NBL1 LMNA FMNL3 DDR2 ADAMTS9 APBB2 CTHRC1 SPRED1 DCHS1 SMAD3 COL3A1 LDB2 S1PR1 CYP7B1 CD34 SELP ANO6 RCC2 F2R CSF1R COL18A1 FLRT2 TPCN1 LAMA2 KANK2 PARVA LPAR1 ITGA1 STARD13 ADAMTS1 JUP SELE SLC12A2 HDAC6 HSPB1 RARRES2 BMPR1A PTK7 RHOJ BCL11B PLVAP COL5A1 LOXL2 LAMC1 SULF1 RDX ITGA11 TPM1 CDC42BPA PTPRG PHLDB2 ADAMTS12 DST DAB2 PDPN CLASP2 INSR MCC LRRC15 EVL EPHA3 TGFBR3 FAT1 GPC3 SPOCK1 EMC10 TAOK2 ROBO4 ID1 MAP1B ESAM JAM2 COL1A2 CDK1 PROS1 |
| D | 8.53E-17 | 30 | Cytokine-mediated signaling pathway | CCL7 CCL2 CCL8 CXCL6 CXCL9 CXCL3 CXCL5 CXCL10 CCL11 CCL19 CCL13 CCL23 WNT5A CCR2 EREG IL7R MMP12 CSF3 IL11 GBP1 CD80 MT2A FGF2 MMP3 IL24 CSF2 TNFRSF11B SAA1 MMP1 HLA-DQA2 |
| D | 1.22E-16 | 26 | Regulation of signaling receptor activity | SERPINE1 PTHLH IL11 CSF3 CCL7 CCL2 CCL8 WNT5A SPP1 CXCL6 EREG FGF2 CXCL9 UCN2 STC1 IL24 CXCL3 CXCL5 CSF2 TNFRSF11B GREM1 CXCL10 CCL11 CCL19 CCL13 CCL23 |
| D | 1.22E-16 | 34 | Response to cytokine | CCL7 CCL2 CCL8 CXCL6 CXCL9 CXCL3 CXCL5 CXCL10 CCL11 CCL19 CCL13 UBD CCL23 CSF3 WNT5A GBP1 CCR2 EREG CSF2 CHI3L1 IL24 IL7R TYMS MMP12 MT2A HAS2 IL11 CD80 FGF2 MMP3 TNFRSF11B SAA1 MMP1 HLA-DQA2 |
| D | 8.25E-16 | 32 | Cellular response to cytokine stimulus | CCL7 CCL2 CCL8 CXCL6 CXCL9 CXCL3 CXCL5 CXCL10 CCL11 CCL19 CCL13 CCL23 CSF3 WNT5A GBP1 CCR2 EREG CSF2 CHI3L1 IL24 IL7R MMP12 MT2A HAS2 IL11 CD80 FGF2 MMP3 TNFRSF11B SAA1 MMP1 HLA-DQA2 |
| D | 1.29E-15 | 18 | Leukocyte chemotaxis | CCL7 CCL2 CCL8 CXCL6 CXCL9 CXCL3 CXCL5 CXCL10 CCL11 CCL19 CCL13 CCL23 CCR2 SAA1 GREM1 GPR183 SERPINE1 WNT5A |
| D | 8.61E-15 | 19 | Cell chemotaxis | CCL7 CCL2 CCL8 CCR2 CXCL6 CXCL9 CXCL3 CXCL5 CXCL10 CCL11 CCL19 SAA1 CCL13 CCL23 FGF2 GPR183 GREM1 SERPINE1 WNT5A |
| D | 3.45E-14 | 13 | Chemokine-mediated signaling pathway | CCL7 CCL2 CCL8 CXCL6 CXCL9 CXCL3 CXCL5 CXCL10 CCL11 CCL19 CCL13 CCL23 CCR2 |
| D | 4.96E-14 | 12 | Lymphocyte chemotaxis | CCL7 CCL2 CCL8 CCL11 CCL19 CCL13 CCL23 CXCL10 SAA1 CCR2 GPR183 WNT5A |
| D | 6.62E-14 | 40 | Response to external stimulus | CCL7 CCL2 CCL8 CD80 CCR2 CXCL6 CXCL9 UCN2 CXCL3 CXCL5 CXCL10 CCL11 CCL19 SAA1 CCL13 CCL23 SERPINE1 SPP1 FGF2 GPR183 MGST1 TRPA1 CSF3 WNT5A GBP1 MT2A IDO1 MMP7 STC1 RCAN1 IL24 CSF2 TNFRSF11B GREM1 TYMS MMP12 CHI3L1 HMGA2 ARNTL2 MMP3 |
| D | 1.00E-13 | 46 | Response to organic substance | CCL7 CCL2 CCL8 SCGB2A2 CD80 CXCL6 CXCL9 UCN2 CXCL3 CXCL5 CXCL10 CCL11 CCL19 CCL13 UBD CCL23 MGST1 CSF3 WNT5A GBP1 SPP1 CCR2 EREG FGF2 CSF2 GREM1 HAS2 TRPA1 MMP19 IDO1 CHI3L1 SLC7A11 STC1 IL24 LY6D IL7R TYMS MMP12 MT2A SERPINE1 IL11 MMP3 TNFRSF11B SAA1 MMP1 HLA-DQA2 |
| D | 1.00E-13 | 13 | Neutrophil chemotaxis | CCL7 CCL2 CCL8 CXCL6 CXCL9 CXCL3 CXCL5 CXCL10 CCL11 CCL19 CCL13 CCL23 SAA1 |
| D | 1.04E-13 | 16 | Myeloid leukocyte migration | CCL7 CCL2 CCL8 CXCL6 CXCL9 CXCL3 CXCL5 CXCL10 CCL11 CCL19 CCL13 CCL23 CCR2 SAA1 GREM1 SERPINE1 |
| D | 2.14E-13 | 20 | Leukocyte migration | CCL7 CCL2 CCL8 CXCL6 CXCL9 CXCL3 CXCL5 CXCL10 CCL11 CCL19 CCL13 CCL23 CCR2 SAA1 GREM1 GPR183 SERPINE1 WNT5A SLC7A11 MMP1 |

Supplementary TABLE S3 The top 10 enriched biological terms in up-regulated genes with input genes

| **Term** | **P-value** | **Genes** |
| --- | --- | --- |
| Immune System_Homo sapiens_R-HSA-168256 | 5.21E-21 | IFITM1;IL1RN;CSF3R;IFITM2;NCF2;WIPF1;UBE2D2;NCF4;ICAM3;ACTB;ACTG1;PYCARD;CFL1;CISH;PRKCB;SLC11A1;PRKCD;HLA-C;CYBA;CNPY3;HLA-A;HLA-E;TYROBP;PRKAR1A;PSME1;TXNIP;CLEC4E;UBA52;ANAPC13;PSMD13;C5AR1;LY96;CSF2RB;SOCS3;UBC;ATP6V1H;CD14;LYN;VASP;STAT5B;SIGLEC14;TNFSF14;UBE2B;DEFA3;ARPC4;ISG15;DEFA1;ARPC5;ISG20;NFKBIA;SELL;ARPC3;GRB2;MYD88;CD22;GSK3B;ARF1;SH3KBP1;ARPC1B;ITGB2;PTEN;BRK1;PIK3CD;ADAR;TREM1;FCGR3A;PSTPIP1;AMICA1;VAV3;DUSP2;HSP90AA1;SYK;DUSP1;IFNGR2;IL16;FOS;KIF22;PPP2R5C;LAT2;HCK;SIKE1;UBE2R2;IL1B;ATP6V1B2;IRF8;LCP2;TLR4;ATP6V0C;MGRN1;DCTN3;WAS;NOD2;FYB;LILRA5;RELB;PAK1;PSMB3;ADRBK1;TCEB2;RNF130;PAK2;OSM;AP2B1;SIGLEC5 |
| Innate Immune System_Homo sapiens_R-HSA-168249 | 8.58E-16 | GSK3B;WIPF1;ARPC1B;UBE2D2;ITGB2;PTEN;BRK1;PIK3CD;ICAM3;TREM1;ACTB;ACTG1;PYCARD;FCGR3A;PSTPIP1;CFL1;VAV3;DUSP2;HSP90AA1;SYK;DUSP1;PRKCD;HLA-C;FOS;CNPY3;PPP2R5C;HLA-E;LAT2;HCK;SIKE1;TYROBP;PRKAR1A;IL1B;PSME1;TXNIP;LCP2;CLEC4E;UBA52;TLR4;PSMD13;C5AR1;WAS;LY96;CSF2RB;NOD2;RELB;PAK1;PSMB3;ADRBK1;UBC;CD14;PAK2;LYN;SIGLEC14;DEFA3;ARPC4;ISG15;DEFA1;ARPC5;NFKBIA;ARPC3;GRB2;MYD88 |
| Signaling by Rho GTPases_Homo sapiens_R-HSA-194315 | 5.10E-15 | HIST2H2AA3;ARHGAP9;WIPF1;NCF2;ARPC1B;NCF4;HIST1H2BK;WAS;BRK1;IQGAP2;ARHGAP15;ACTB;HIST2H2AC;ACTG1;PAK1;ARHGDIB;TAGAP;RAC2;PAK2;HIST1H2AC;VAV3;NCOA2;H2AFZ;NDE1;H3F3A;RHOG;HMHA1;CYBA;ARPC4;ARPC5;PPP2R5C;GMIP;ARHGAP25;HIST2H2BE;RHOB;MYL6;ARPC3;GRB2;PFN1;HIST1H2BC |
| RHO GTPase Effectors_Homo sapiens_R-HSA-195258 | 6.01E-14 | HIST2H2AA3;WIPF1;NCF2;ARPC1B;NCF4;HIST1H2BK;WAS;BRK1;IQGAP2;ACTB;HIST2H2AC;ACTG1;PAK1;RAC2;PAK2;HIST1H2AC;NCOA2;H2AFZ;NDE1;H3F3A;RHOG;CYBA;ARPC4;ARPC5;PPP2R5C;HIST2H2BE;RHOB;MYL6;ARPC3;GRB2;PFN1;HIST1H2BC |
| Cellular responses to stress_Homo sapiens_R-HSA-2262752 | 6.21E-13 | HIST2H2AA3;GSK3B;ANAPC16;MRPL18;UBE2D2;HIST1H2BK;WDR45;GABARAP;HIST2H2AC;LMNB1;DNAJB1;PRDX5;UBC;TCEB2;HIST1H1E;HIST1H2AC;HIST1H1C;CDKN2D;KDM6B;EGLN3;HSP90AA1;H2AFZ;TINF2;H3F3A;HSPA6;CYBA;FOS;HIST2H2BE;TFDP1;CAT;CHMP4A;LAMTOR1;LAMTOR4;UBA52;HSPA1B;HSPA1A;HIST1H2BC |
| Fcgamma receptor (FCGR) dependent phagocytosis_Homo sapiens_R-HSA-2029480 | 1.47E-10 | LYN;VAV3;HSP90AA1;SYK;WIPF1;ARPC1B;PRKCD;WAS;BRK1;ARPC4;ARPC5;ACTB;ACTG1;HCK;FCGR3A;PAK1;ARPC3;CFL1;GRB2 |
| EPH-Ephrin signaling_Homo sapiens_R-HSA-2682334 | 1.89E-10 | VAV3;LYN;HSP90AA1;ARPC1B;ARPC4;AP2B1;ARPC5;ACTB;ACTG1;SDCBP;MYL6;PAK1;NCSTN;ARPC3;CFL1;EPHB1;PAK2 |
| Adaptive Immune System_Homo sapiens_R-HSA-1280218 | 5.16E-10 | GSK3B;ARF1;IFITM1;NCF2;SH3KBP1;UBE2D2;NCF4;ITGB2;PTEN;PIK3CD;ICAM3;TREM1;FCGR3A;AMICA1;SYK;PRKCB;HLA-C;CYBA;HLA-A;KIF22;PPP2R5C;HLA-E;TYROBP;UBE2R2;PSME1;LCP2;UBA52;ANAPC13;MGRN1;PSMD13;DCTN3;WAS;FYB;LILRA5;SOCS3;PAK1;PSMB3;UBC;TCEB2;RNF130;PAK2;LYN;VASP;UBE2B;AP2B1;NFKBIA;SELL;GRB2;CD22;SIGLEC5 |
| Regulation of actin dynamics for phagocytic cup formation_Homo sapiens_R-HSA-2029482 | 2.26E-09 | VAV3;HSP90AA1;SYK;WIPF1;ARPC1B;WAS;BRK1;ARPC4;ARPC5;ACTB;ACTG1;FCGR3A;PAK1;ARPC3;CFL1;GRB2 |
| C-type lectin receptors (CLRs)_Homo sapiens_R-HSA-5621481 | 1.15E-08 | LYN;SYK;PSMD13;UBE2D2;PRKCD;ICAM3;RELB;PYCARD;NFKBIA;PAK1;PSMB3;IL1B;UBC;PSME1;CLEC4E;UBA52;PAK2 |

Supplementary TABLE S4 The top 10 enriched biological terms in down-regulated genes with input genes

| **Term** | **P-value** | **Genes** |
| --- | --- | --- |
| Gene Expression_Homo sapiens_R-HSA-74160 | 1.20E-04 | NUP107;NUP188;PHAX;MTO1;NARS;IPO8;SIN3B;ZNF528;SNAPC3;ZNF17;ZNF562;ZNF484;PRKAB2;NCBP1;EED;TYW3;TOP3A;TSC1;PLRG1;THOC2;DDX52;BAZ1B;MED23;VWA9;SCO1;MTF2;POLR1C;ZNF559;ZNF436;SRSF6;ZNF510;ZNF432;SKIV2L2;ZNF431;YAP1;RPN2;GTF3C6;HEATR1;TARS;NR2C1;RPAP2;NUP160;WRN;NUP85;POLR2F;NUP88;ZNF227;ZNF302;ZNF585B;RPP14;ZNF189;SF3A3;EIF2B5;ZNF141;ZNF460;WDR12;RCL1;SNRNP40;ERCC3;ZNF736;TRMT13;SRPRB;RBMX;TNRC6A;COX20 |
| Transport of the SLBP independent Mature mRNA_Homo sapiens_R-HSA-159227 | 1.48E-04 | NUP107;NUP188;NCBP1;NUP85;NUP88;NUP160 |
| Transport of the SLBP Dependant Mature mRNA_Homo sapiens_R-HSA-159230 | 1.76E-04 | NUP107;NUP188;NCBP1;NUP85;NUP88;NUP160 |
| Metabolism of non-coding RNA_Homo sapiens_R-HSA-194441 | 2.31E-04 | NUP107;NUP188;NCBP1;NUP85;PHAX;NUP88;NUP160 |
| snRNP Assembly_Homo sapiens_R-HSA-191859 | 2.31E-04 | NUP107;NUP188;NCBP1;NUP85;PHAX;NUP88;NUP160 |
| Transport of Mature mRNA Derived from an Intronless Transcript_Homo sapiens_R-HSA-159231 | 2.85E-04 | NUP107;NUP188;NCBP1;NUP85;NUP88;NUP160 |
| Transport of Mature mRNA derived from an Intron-Containing Transcript_Homo sapiens_R-HSA-159236 | 2.86E-04 | NUP107;NUP188;NCBP1;NUP85;NUP88;THOC2;SRSF6;NUP160 |
| Transport of Mature mRNAs Derived from Intronless Transcripts_Homo sapiens_R-HSA-159234 | 3.31E-04 | NUP107;NUP188;NCBP1;NUP85;NUP88;NUP160 |
| Transcriptional regulation by small RNAs_Homo sapiens_R-HSA-5578749 | 4.25E-04 | IPO8;NUP107;NUP188;NUP85;POLR2F;NUP88;NUP160;TNRC6A |
| Transport of Mature Transcript to Cytoplasm_Homo sapiens_R-HSA-72202 | 5.12E-04 | NUP107;NUP188;NCBP1;NUP85;NUP88;THOC2;SRSF6;NUP160 |
